# Transplant-Associated Thrombotic Microangiopathy in the Context of Allogenic Hematopoietic Stem Cell Transplantation: Where We Stand

**DOI:** 10.3390/ijms24021159

**Published:** 2023-01-06

**Authors:** Ioanna Lazana

**Affiliations:** 1 Cell and Gene Therapy Laboratory, Biomedical Research Foundation of the Academy of Athens, 11527 Athens, Greece; ilazana@doctors.org.uk; 2 Hematology Department, King’s College Hospital NHS Foundation Trust, London SE5 9RS, UK

**Keywords:** transplant-associated thrombotic microangiopathy, allogenic hematopoietic stem cell transplantation, complement inhibitors

## Abstract

Transplant-associated thrombotic microangiopathy (TA-TMA) constitutes a significant contributor to the increased morbidity and mortality after allogenic hematopoietic stem cell transplantation (allo-HSCT). TA-TMA is a heterogenous disease, characterized by the triad of endothelial cell activation, complement dysregulation and microvascular hemolytic anemia, which may affect all organs. The lack of consensus diagnostic criteria, along with the common clinical features mimicking other diseases that complicate allo-HSCT, make the diagnosis of TA-TMA particularly challenging. Significant effort has been made to recognize specific risk factors predisposing to the development of TA-TMA and to identify serum biomarkers predicting the development of the disease. With regard to treatment, therapeutic plasma exchange (TPE) has been traditionally used, although with doubtful efficacy. On the other hand, the pivotal role of complement activation in the pathophysiology of TA-TMA has led to the exploration of the therapeutic potential of complement inhibitors in this setting. Eculizumab has been proposed as a first-line therapeutic agent in TA-TMA, owing to the very promising results in both pediatric and adult clinical trials. Pharmacokinetic and pharmacodynamic studies and CH50 levels are of paramount importance in the allo-HSCT setting, as a different dosing schedule (more intensive—in dose and frequency—at the beginning) seems to be required for successful outcomes. Furthermore, Narsoplimab, a MASP-2 inhibitor, recently received a Breakthrough Therapy Designation from the FDA for the treatment of TA-TMA after allo-HSCT. Finally, the decision to withdraw the CNIs, although initially advised by the Bone and Marrow Transplant Clinical Trials Network Committee, remains debatable owing to the controversial results of recent clinical trials. This review summarizes the current updates on pathophysiology, diagnosis and therapeutic approaches and emphasizes future goals and perspectives.

## 1. Introduction

Allogenic hematopoietic stem cell transplantation (allo-HSCT) remains the only curative option for many malignant and non-malignant diseases, with over 50,000 allo-HSCTs performed annually worldwide [1]. Despite the improvements in allo-HSCT outcomes in the last decade, owing to better supportive care, modification of the conditioning regimen, choice of immunosuppression, etc., increased morbidity and mortality remain a significant issue, restricting its broader application [2]. Contributing factors include the occurrence of (acute and late) graft-versus-host disease (GvHD), disease relapse, infections, etc. [3,4]. Thrombotic microangiopathy (TMA) following allo-HSCT, termed transplant-associated thrombotic microangiopathy (TA-TMA), has been increasingly recognized as a significant risk factor for increased morbidity and mortality after allo-HSCT [5,6,7].

TA-TMA is characterized by microangiopathic hemolytic anemia and thrombocytopenia, as a result of generalized endothelial dysfunction and complement activation, leading to microthrombi formation [8]. It has a very heterogenous clinical picture and may affect any organ of the body but most commonly the kidneys and the brain. Since these features are also shared between other diseases, such as capillary leak syndrome, GvHD, thrombotic thrombocytopenic purpura (TTP), veno-occlusive disease (VOD), etc., the diagnosis of TA-TMA is commonly delayed or masked. This, along with the lack of consensus criteria for TA-TMA diagnosis, results in delayed or inappropriate treatment, leading to the increased morbidity and mortality associated with the syndrome [9]. However, in recent years, there has been an increased effort to better define the diagnostic criteria for TA-TMA and validate them in prospective studies. Furthermore, there has been huge progress in elucidating the pathophysiology of the disease, which would also facilitate better therapeutic approaches. This review summarizes the current updates on the pathophysiology, diagnosis and therapeutic approaches of TA-TMA and cites future goals and perspectives.

## 2. Pathophysiology of TA-TMA

TA-TMA belongs to a spectrum of transplant-associated endothelial cell activation syndromes, including capillary leak syndrome, idiopathic pneumonia syndrome, engraftment syndrome, etc. [10]. It is a systemic, heterogeneous disease, which is characterized by the triad of endothelial cell activation, complement dysregulation and microvascular hemolytic anemia, and it may affect all organs. Those features are also common in other diseases, such as atypical hemolytic uremic syndrome (aHUS) and thrombotic thrombocytopenia purpura (TTP), making the diagnosis challenging.

With regard to pathophysiology, a three-hit theory has been recently suggested by Luft et al. [11]. This includes: (1) An underlying predisposition to complement activation or endothelial injury (endothelial vulnerability) (hit 1). This implies that pre-existing defects may affect the response of endothelium and/or complement eventual challenges occurring after allo-HSCT. This is based on the observations that specific single nucleotide polymorphisms (SNPs) in recipient genes or raised plasma markers associated with endothelial cell distress prior to allo-HSCT are associated with poor outcomes in patients with GvHD, without having any impact on patients who did not develop GvHD [12,13]. (2) A toxic event (such as the conditioning regimen), leading to the secretion of proinflammatory cytokines and procoagulant proteins and the loss of protective mechanisms (such as depletion of nitric oxide and vascular endothelial growth factor), that cause endothelial injury and initiate the complement cascade (hit 2). (3) Additional insults (such as infections, drugs/immunosuppressants, etc.) propagate the complement activation, leading to the subsequent microthrombi formation (hit 3).

## 3. Incidence, Risk Factors and Outcome of TA-TMA

Although the association between HSCT and TMA is well-documented [14,15], the precise incidence of TA-TMA remains largely unknown, varying between 0.5% and 76% in the allo-HSCT setting, whereas its incidence is slightly lower in the autologous setting (0–27%) [8,16]. This is partly due to the lack of a gold standard for its diagnosis, leading to variable definitions of TA-TMA in different centers and studies.

TA-TMA is most often described as an early event in allo-HSCT with a time of onset between 32 and 86 days [17,18], although a recent study by Heybeli et al. [19] documented a bimodal distribution of TA-TMA, with a first peak at day 27 and a second peak around day 200.

TA-TMA is considered one of the most severe complications of allo-HSCT [6]. Prognosis is generally poor with a case-fatality varying between 50 and 75% [6,19], underlying the need for early prognostic markers to be defined. The role of renal impairment in predicting lower survival rates has been well documented [18,20,21]. Jodele et al. [18] identified proteinuria (>30 mg/dL) as a significant predictive factor for survival. More specifically, patients with proteinuria and elevated C5–9 levels had a 1-year survival of <20%, whereas patients with normal serum C5–9 levels and no proteinuria had a survival of 100%. Although very well designed and prospective, this study was not powered to examine the outcome of death from TA-TMA, and future randomized studies are required to validate this finding. Another study by Cho et al. [21] identified renal dysfunction and concurrent VOD as the only factors associated with shorter overall survival (OS) and higher transplant-related mortality (TRM), but this was a univariate analysis and should be interpreted with caution. Finally, the Endothelial Activation and Stress Index (EASIX) has been proposed to predict mortality in TA-TMA [22]. EASIX has emerged as a useful, dynamic biomarker, reflecting the degree and progression of endothelial damage in post-allo-HSCT patients. It is calculated using the formula: lactate dehydrogenase (U/L) × creatinine (mg/dL)/thrombocytes (10^9^ cells/L), on days 0, 30, 100 and at last follow-up. Although very promising, these findings carry significant limitations, such as low cohort numbers, failure to assess the effect of the conditioning therapy on endothelial function and complement activation and the difference in common time-points of EASIX calculation chosen by the investigators.

Given the significantly reduced survival in TA-TMA patients, the recognition and prevention (if possible) of the risk factors for its development remain essential. Several pre-transplant risk factors have been found to be independently associated with the development of TA-TMA in multivariable analyses, such as age, mismatched donor transplantation, myeloablative (total body irradiation-based) conditioning, non-malignant disorders and history of prior allo-HSCT [23]. Post-transplant risk factors include the development of acute GvHD (aGVHD, constitutes the strongest risk factor, ×4-fold increase), positive CMV serostatus, high disease risk index, high baseline LDH level, elevated Calcineurin-inhibitor (CNI) levels, mammalian target of rapamycin inhibitors (mTORi), venous thromboembolic disease and infections (bacteremia, invasive aspergillosis and BK viremia) [16,18,23,24]. Furthermore, a recent study by Gavriilaki et al. [25] reported that Ruxolitinib administration was independently associated with TA-TMA in a retrospective, single-center study of patients with overlap and chronic GvHD. Although the study has several limitations (such as the very low patient numbers and the focus on patients with overlap or chronic GvHD), it reports for the first time that the novel agents used for the treatment of GvHD may have an association with the development or propagation of TA-TMA. Certainly, larger prospective studies are required to confirm this finding.

With regards to serum biomarkers, increased serum levels of the Ba protein early post-allo-HSCT (day 7) have been shown to predict the development of TA-TMA and non-relapse mortality (NRM) in a single-center, retrospective study [26]. Factor Ba is a fragment of the complement factor B, which is an essential component of the alternative pathway of the complement activation system. Other studies have suggested that elevated plasma levels of complement activation proteins (such as C5b-9 and BBPLus) at the onset or 2–4 weeks after the onset of aGvHD are high-risk factors for the development of TA-TMA [27]. Finally, elevated serum neutrophil extracellular traps (NETs) either on day 0 (prior to conditioning) or 4 weeks after allo-HSCT were associated with an increased risk of developing TA-TMA, whereas thrombomodulin had no predictive impact in a small retrospective study of 11 patients with TA-TMA [28]. Larger, randomized studies are required for validation in all cases.

Based on the above, a number of medications, such as statins, ursodeoxycholic acid (UDA) and defibrotide, have been explored in an effort to prevent the development or evolution of endothelial damage and TA-TMA [11]. Statin prophylaxis, with or without the concomitant use of UDA, has been shown to be safe and to reduce the risk of TA-TMA, thus improving survival [29]. More specifically, statins, beyond their lipid-lowering activity, have been shown to have other beneficial effects, such as immunological actions (suppression of MHC-II-mediated T-cell activation and T-cell cytotoxicity, down-regulation of dendritic cell-derived IFN-a production, etc.) and preservation of endothelial function [29], whereas UDA, beyond the protective effect on liver parenchymal cells, has been shown to have immunomodulatory effects, to activate the glucocorticoid receptor and to repress the release of inflammatory cytokines [30]. This has led some transplant centers to introduce statin-based prophylaxis from endothelial injury, combining UDA and pravastatin [11,31]. The use of defibrotide, with or without the concomitant use of UDA, showed a significant reduction of VOD and aGVHD but had no effect on TA-TMA, as compared to (non-randomized) controls [11,32]. Randomized controlled trials are required to validate the beneficial effect of UDA and statins and potentially identify other medications to protect the injured endothelium.

A possible link between genetic variants in complement regulatory proteins (CRPs) has also been suggested [33]. More specifically, it has been demonstrated that donor-derived cells, bearing a CRP variant, caused impaired regulation of complement activation, as was illustrated by the increased numbers of HSCT-TMA, suggesting that the donors could potentially be screened for these variants prior to allo-HSCT. However, this involved a very small cohort study of 16 patients only. Furthermore, the diagnosis of TA-TMA was based only on thrombocytopenia, non-immune hemolysis and renal injury, without excluding other causes such as disseminated intravascular coagulation (DIC), TTP, aHUS, etc. Another link between endothelial injury and complement activation was provided by Gloude et al. [34], who observed increased levels of circulating double-strand DNA (dsDNA), a known surrogate for neutrophil extracellular traps (NETs), around the time of engraftment. This increase in dsDNA was found to be strongly associated with an increased risk of later TA-TMA, suggesting that early complement activation may initiate later adverse clinical outcomes. Interestingly, the dsDNA levels normalized after treatment with complement inhibitors in patients without GvHD, whereas an improvement (but not normalization) was noticed in patients with ongoing GvHD, suggesting an ongoing endothelial injury driven by GvHD. Despite this very interesting observation, it should be noted that: (i) this was a pediatric cohort, with the majority of children being transplanted for nonmalignant diseases, so different results may be seen in adults transplanted for malignant disorders, (ii) the complement levels were not measured and (iii) dsDNA is only a surrogate marker for NETs and, in the context of wide tissue inflammation/injury, may be proven inaccurate. Finally, Balassa et al. [35] investigated whether HLA-DR1*11 could predispose to TA-TMA, having a role in its pathogenesis. This hypothesis was based on the recognized association between HLA-DR1*11 and idiopathic TTP [36]. This single-center, retrospective study of 425 allo-HSCT patients proposed a strong association between HLA-DR1*11 and the development of TA-TMA. More interestingly, the authors observed significantly better survival in TA-TMA-affected patients carrying HLA-DR1*11 compared to those who did not, but the authors failed to provide an explanation for this observation. The study was also limited by the retrospective nature of the analysis and the relatively small cohort (only 18% of patients had TA-TMA).

## 4. Diagnostic Parameters for TA-TMA

TA-TMA is broadly characterized by microangiopathic hemolytic anemia (in the absence of coagulopathy), hypertension and organ dysfunction (mainly renal, but also central nervous system, intestinal, etc.) [8]. Since 2005, there has been a significant effort to develop consensus criteria for TA-TMA diagnosis. The Blood and Marrow Transplant Clinical Trials Network (BMT-CTN) [37] and the International Working Group (IWG) [38] proposed a different set of criteria for diagnosing TA-TMA in the allo-HSCT setting. More specifically, the BMT-CTN suggested the following four criteria for TA-TMA diagnosis: (1) RBC fragmentation with ≥2 schistocytes per high-power field on a peripheral smear; (2) increased serum lactate dehydrogenase (LDH) above baseline; (3) concurrent renal and/or neurologic dysfunction without other explanations and (4) negative direct and indirect Coombs test results, whereas the IWG included the following five criteria: (1) >4% schistocytes in blood; (2) de novo thrombocytopenia (platelet count < 50 × 109/L or 50% or greater reduction from previous counts); (3) sudden and persistent increased LDH; (4) decreased hemoglobin or increased transfusion requirement and (5) decreased haptoglobin. A validation study by Cho et al. [21] compared the two in an analysis of 672 allo-HSCT patients. The authors concluded that both definitions carried significant weaknesses and pitfalls, such as the lack of exclusion criteria for DIC, the absence of renal involvement (which was found to be associated with poor survival) or the non-inclusion of thrombocytopenia and the lack of sensitivity. In a subsequent study by Shayani et al. [39], the authors divided the patients into those with ‘possible’ and those with ‘probable’ TA-TMA diagnosis, based on the City of Hope criteria (Table 1). The study included 177 patients, of which 17% were found to have possible and 22% probable TA-TMA. Postalcioglu et al. [7] used the same City of Hope criteria in a retrospective study of nearly 2000 adult allo-HSCT patients, and they identified 13% with definite and 26% with probable TA-TMA. It is of note that only 17% were clinically diagnosed with TA-TMA, underlying the significant under-recognition of this syndrome, which translates to high morbidity and mortality, due to a lack of prompt and appropriate therapeutic intervention(s). Finally, Jodele et al. [18] attempted to define ‘high-risk TA-TMA’ in a prospective pediatric cohort of 100 patients. More specifically, the authors observed that the combination of proteinuria (>30 mg/dL) and elevated markers of complement activation (C5b-9) were associated with very high mortality (NRM 84% at 1-year post-allo-HSCT). This definition has been recently updated to include patients with multi-organ failure [40]. More specifically, high-risk diseases also included patients with the presence of one of the two aforementioned high-risk features and clinical evidence of multi-organ failure. This is of importance as the identification of high-risk diseases affected treatment decisions, leading to improved outcomes. Although very important, it has to be noted that the study is restricted to a pediatric population, mainly with immune deficiencies, limiting its translation to the adult population with underlying malignancies, where the complement activation pathways and endothelial homeostasis may differ [41]. It is, thus, of paramount importance to identify appropriate disease-defining markers for TA-TMA diagnosis, although the functional heterogeneity of the disease should always be kept in mind when attempting to define diagnostic criteria for TA-TMA.

## 5. Clinical Presentation

TA-TMA can occur in multiple organs resulting in a variable clinical picture, including intestinal TMA (iTMA), pulmonary hypertension, posterior reversible encephalopathy syndrome and multiple organ failure [24,42]. This heterogenous presentation, however, resembles other complications of allo-HSCT, implying that a high index of clinical suspicion is required for the prompt and correct diagnosis of TA-TMA.

The most commonly affected organs are:

(i) Kidneys: They constitute the primary target of TA-TMA, leading to proteinuria, hypertension and renal failure. Their involvement is associated with poor prognosis, highlighting the need for early recognition and treatment initiation [24]. Although tissue biopsy constitutes the gold standard for diagnosis, this may be precluded in the early post-allo-HSCT period due to high bleeding risk, so other markers predicting renal involvement are required. Hypertension is a well-documented predictive factor that can be easily monitored [18]. Screening for TA-TMA is recommended for patients with persistent or refractory hypertension (requiring more than two anti-hypertensives) post-allo-HSCT. Similarly, proteinuria is an established marker of renal dysfunction and a marker of severe disease, associated with increased mortality, that can be easily measured [43,44]. Elevated creatinine levels, on the other hand, have poor predictive potential, as they have low sensitivity in certain sub-populations and occur late in the course of TA-TMA [18].

(ii) Gastrointestinal tract (GI): GI TA-TMA is also a well-recognized and common complication of TA-TMA, associated with high-risk features [45,46]. It shares the same clinical (abdominal pain, diarrhea, ascites and GI bleeding) and histological features with other common complications of allo-HSCT (such as colitis, GI GvHD and infections), making the diagnosis particularly difficult [47]. This is of importance as it is now evident that isolated treatment of one pathology (e.g., GvHD) does not improve survival if there is another concomitant underlying pathology (e.g., GI TA-TMA). In the case of GI TA-TMA, Yamada et al. [48] documented a high frequency of GI GvHD (80%) among patients with GI TA-TMA, suggesting a close association between the two pathologies, whereas Inamoto et al. [49] observed that GI TA-TMA was responsible for 57% of NRM in patients with persistent GI symptoms. Although these studies are retrospective in nature and lack consensus criteria, they clearly highlight that increased vigilance is required for prompt diagnosis of GI TA-TMA, irrespective of the presence of other confirmed diagnoses (such as GI GvHD and CMV colitis), in order to improve outcomes.

(iii) Central Nervous System (CNS): The CNS’s involvement is also associated with an unfavorable prognosis in TA-TMA [8]. It may present as headache, confusion and seizures, whereas posterior reversible encephalopathy has been described in the pediatric population in association with uncontrolled hypertension [50].

(iv) Pulmonary: Pulmonary hypertension (PH) is the hallmark of pulmonary TA-TMA but it lacks specificity [51]. It most commonly presents as unexplained hypoxemia, pleural effusion and respiratory distress and is associated with high morbidity and mortality [52]. Although cardiac catheterization remains the gold standard in the diagnosis of PH, its application is restricted in the allo-HSCT setting due to the high procedure risk and the significant patient comorbidities.

(v) Multi-organ dysfunction: TA-TMA, as an endothelial dysfunction disorder, may affect any part of the vasculature, leading to multi-organ dysfunction. Naturally, it is associated with very high morbidity and mortality, requiring intensive care support and a multidisciplinary management approach [24], highlighting once more that a high-suspicion index is required for accurate and prompt diagnosis and installation of the appropriate (combination of) treatment.

## 6. Current Therapeutic Approaches

Recent advances in elucidating the pathophysiology of TA-TMA have allowed better and broader therapeutic management of patients with TA-TMA, in a multidisciplinary approach.

### 6.1. Preventative Measures

A number of pre- and post-transplant risk factors have been identified to contribute to the development of TA-TMA, allowing the design of a preventative strategy to minimize the risk of developing TA-TMA. Such preventative measures include HLA-matched transplantation, avoidance of endothelial toxins (such as mTOR inhibitors), use of conditioning regimens with a reduced intensity that may minimize endothelial injury and, most importantly, vigorous infection control [41]. This is of particular importance as patients who are considered to be low-risk for TA-TMA may entirely avoid the development of TA-TMA using a simple implementation of preventative measures. Even in high-risk patients, the application of preventative measures may delay or minimize the degree of complement activation and endothelial damage. This is evident indirectly by the improved outcomes in the last decade, where higher vigilance for TA-TMA exists and preventative measures have been implemented [53].

### 6.2. Supportive Care

The role of supportive care in the management of TA-TMA is of paramount importance. This involves: minimizing transfusions, aggressive management of hypertension, prompt and aggressive treatment of co-existing infection(s), renal replacement therapy, etc. [24,53].

### 6.3. Therapeutic Interventions

The therapeutic interventions include:

(i) Withdrawal of offensive medications (such as CNIs):

It is now evident that CNIs contribute to the development of TA-TMA, by increasing the synthesis of thromboxane A2, while decreasing the production of prostacyclin and inhibiting nitric oxide synthase (NOS), thus disrupting endothelial homeostasis [54,55,56]. On this basis, the Blood and Marrow Transplant Clinical Trials Network Toxicity Committee consensus advised the prompt discontinuation of CNIs as the primary intervention for TA-TMA management [37]. However, in a large, single-center, retrospective study by Li et al. [23] discontinuation of CNIs failed to improve survival, irrespective of the type of GvHD prophylaxis. This may be attributed to a subsequent development or exacerbation of GvHD, which is not only related to increased morbidity and mortality per se, but also constitutes one of the main triggers of TA-TMA. Withdrawal of CNIs should, thus, be performed with caution. With regards to the appropriate alternative to CNIs for GvHD prophylaxis, Wolff et al. [57] explored the potential use of daclizumab, an IL-2 inhibitor. The results showed that 9/13 patients achieved complete remission (CR) of TA-TMA, 2/13 had stable disease and 1/13 did not respond. However, despite the very encouraging results, the mortality remained high in patients with aGvHD, with a median survival of 39 days. This underlines that further studies are warranted to explore substitution strategies, potentially with the use of novel agents, such as JAK2 inhibitors.

(ii) Therapeutic Plasma Exchange (TPE): Historically, TPE had been the mainstay of treatment for TA-TMA, based on the experience and life-saving results in TTP [58]. However, it has now been established that TPE should not be considered a standard of care for TA-TMA. This was mainly based on the observation of low response rates of 37% and high mortality rates of 79% in a group of 121 TPE-treated patients [37]. In another study by Fuge at al. [59], response to TPE was noted in 6/17 TA-TMA patients. However, only one of those six patients survived, resulting in a 6% success rate. A similarly low success rate was also documented by Roy et al. [60], with 4/17 patients responding to TPE but only 1/4 surviving. Moreover, the complication rates of TPE are significant and cannot be ignored [61,62]. The discrepancy in the therapeutic results of TPE between TTP and TA-TMA may be related to the different pathophysiology of TA-TMA, having normal ADAMTS13 levels.

(iii) Defibrotide: The protective effects of Defibrotide on endothelium, reducing the procoagulant activity and increasing the fibrinolytic properties of endothelial cells, are well-documented [63]. In addition, it was recently shown to protect the endothelium from the harmful effects of cyclosporine and tacrolimus or tacrolimus combined with sirolimus [64]. Defibrotide, which is considered the gold-standard therapy for VOD, has also been explored in the TA-TMA setting with encouraging results. In a multicenter retrospective study, the use of Defibrotide for the treatment of TA-TMA was associated with a favorable outcome based on a univariate analysis with 30/32 patients, who were alive at 180 days from the onset of TA-TMA, being treated with Defibrotide (4 in combination with TPE). Furthermore, no major adverse events were noted during Defibrotide treatment [14]. Another study explored the potential effect of a low dose of Defibrotide on TA-TMA in a very limited number of patients and demonstrated that Defibrotide was able to induce remission in all cases, even at a lower dose [65]. Along the same lines, Yeates et al. reported resolution of TA-TMA symptoms in 76% of a small cohort of 17 patients [66], whereas Bohl et al. reported a response rate of 61% in patients treated with Defibrotide with or without TPE and/or Rituximab [67]. Finally, a pilot phase II study investigated the potential prophylactic use of Defibrotide in pediatric patients. More specifically, Defibrotide was used during conditioning and the acute recovery phase (day + 21). The results revealed a reduced incidence of TA-TMA of 4%, compared to the historic rate of 18–40%, suggesting a prophylactic role for Defibrotide in post-allo-HSCT TA-TMA [68]. Certainly, larger prospective studies are required to validate these results, both in pediatric and adult patients requiring allo-HSCT for malignant and non-malignant diseases.

(iv) Anti-complement agents: The pivotal role of complement activation in the pathophysiology of TA-TMA has led to the exploration of the therapeutic potential of complement inhibitors in this setting. Eculizumab, a humanized monoclonal anti-C5 antibody that blocks the terminal pathway of the complement and prevents the formation of the membrane attack complex (MAC), has shown promise in the treatment landscape of TA-TMA. A single-center, retrospective study of 12 patients with severe TA-TMA who were treated with Eculizumab (dosed according to the FDA-approved dosing for atypical hemolytic-uremic syndrome, aHUS), reported a 50% complete response (defined as hematological response and the disappearance of all TMA-associated organ dysfunction) and 33% overall survival, whereas aGvHD was the only factor associated with inferior outcomes [69]. The Eculizumab group was treated with Eculizumab, immunosuppression, therapeutic plasma exchange and/or other supportive care measures. Another study by Jan et al. [70] reported 80% hematologic (defined as normalization of LDH, the disappearance of schistocytes and improvement in transfusion requirements at 4 weeks after initiation of TA-TMA therapy) or complete response (defined as hematologic response with organ recovery) rates with the use of Eculizumab (dosed as in aHUS), compared to 0% in the conventional treatment group. The Eculizumab therapy was accompanied by a change in immunosuppression (from tacrolimus to sirolimus), whereas 7 out of 12 patients received Eculizumab maintenance. OS was 60% in the Eculizumab group versus 30% in the conventional treatment group, supporting the use of Eculizumab as a first-line agent for the treatment of severe TA-TMA. Apart from the retrospective nature of the study and the very small numbers, other limitations include the co-existence of aGvHD and/or infections that could affect mortality, the slightly younger age represented in the Eculizumab group and the failure to measure complement and Eculizumab levels.

The importance of measuring Eculizumab levels and monitoring CH50 levels (total complement activity) was demonstrated in a small pediatric cohort of six children with severe TA-TMA [71]. The use of Eculizumab led to the complete resolution of symptoms in 4/6 patients. It is of note that a dose higher than that recommended for aHUS was used in order to achieve therapeutic levels and secure complete complement inhibition (low CH50). The two patients who failed to achieve therapeutic levels did not survive, suggesting that Eculizumab dosing should be guided using pharmacokinetic (PK) or pharmacodynamic (PD) testing to ensure therapeutic levels are achieved in most ill and catabolic patients, whereas CH50 monitoring may guide dose adjustments in those patients. A prospective study by Jodele et al. [40] exploring the use of Eculizumab in 64 high-risk pediatric patients, documented a response rate of 64% and OS of 66% at 1-year post-HSCT. It is of note that a very different strategy (from that reported in aHUS) was followed, with a brief but intensive course of Eculizumab being given at the beginning of the treatment. This was due to the very high Eculizumab clearance and highly activated complement noted in the HSCT recipients, requiring Eculizumab dosing based on Eculizumab trough and CH50 levels. All the above support the use of Eculizumab as a first-line therapy for TA-TMA, with a different dosing schedule (more intensive—in dose and frequency—at the beginning) based on PK/PD testing and CH50 levels. Successful cessation of Eculizumab treatment has been suggested after full resolution of hematologic parameters and full complement blockade (demonstrated by low CH50 level) [40]. Finally, meningococcal vaccination or prophylactic use of antibiotics has been recommended with the use of complement inhibitors to minimize the risk of infection with encapsulated bacteria (such as Neisseria meningitidis) associated with this type of treatment [72].

(v) Anti-CD20 antibodies (Rituximab): Rituximab has been historically used along with TPE in patients with auto-Abs to ADAMTS13 in TTP and with auto-Abs to complement factor H (CFH) in aHUS, which has shown very good results. In the context of TA-TMA, most of the cases published (12/15) have shown a positive response to Rituximab (either as monotherapy or in combination with TPE or Defibrotide), although the exact mechanism remains unknown [58]. However, the effect of Rituximab on immune regulation is supported by reports of benefit in patients with C4d deposition or CHF auto-Abs after HSCT [73,74].

(vi) MASP-2 inhibition: Narsoplimab, a mannan-binding lectin-associated serine protease-2 (MASP-2), is an inhibitor of the lectin pathway of the complement cascade. Narsoplimab blocks complement activation and endothelial injury without affecting pathways of innate immunity, sparing the immunologic dysfunction noted with other complement inhibitors (such as Eculizumab) [24]. In a recent study of 19 patients with TA-TMA treated with Narsoplimab, survival at day 100 was significantly better compared to historical controls (53% vs. 10%). Disease markers (platelet count, haptoglobin and LDH) also improved in the Narsoplimab group [75]. Despite the limitations of the study (small cohort, no information on the use of immunosuppressants and lack of specific dose regimen), Narsoplimab received a Breakthrough Therapy Designation from the US FDA in 2022 for the treatment of TA-TMA after allo-HSCT.

## 7. Conclusions

Despite the recent advances in understanding the pathophysiology of TA-TMA, many disease aspects remain elusive, such as the diagnostic criteria, the optimal timing of treatment commencement, the appropriate choice of treatment and the relevant dose and treatment schedule, all having a great impact on morbidity and mortality.

It is now well-established that delays in TA-TMA diagnosis may lead to uncontrolled complement activation and endothelial dysfunction with profound implications on survival. As such, there is an imperative need for consensus diagnostic criteria to be developed in order to secure an accurate and prompt disease diagnosis. This would also allow the use of common diagnostic grounds in clinical trial design, allowing for a direct comparison of the respective results and drawing accurate conclusions. Predicting the risk and severity of TA-TMA is also essential for outcomes. However, measurements of serum C5b9 levels and assessment of biological markers of endothelial injury may constitute a limitation in several centers.

The need for large clinical trials on TA-TMA cannot be emphasized enough. However, given the rarity of the disease, this would require the collaboration of multiple centers in order to be able to power the studies. A number of clinical questions will need to be addressed in prospective studies, such as (i) the appropriate treatment modality: validate the efficacy of complement inhibitors, define the appropriate administration scheme and assess the potential added benefit of combined therapy (e.g., with Defibrotide), assess the efficacy of new complement inhibitors (such as long-acting C5 or proximal complement inhibitors), etc.; (ii) the modulation of immunosuppression: is the cessation of CNIs necessary and, if so, what is the most appropriate substitute? and (iii) ascertain the appropriate timing for successful treatment cessation, etc. It has to be noted that most of the currently available studies refer to pediatric populations, so conclusions need to be carefully interpreted and translated when referring to adult patients, as the pathophysiology of the disease varies significantly between the two.

Emerging data have emphasized the importance of performing pharmacokinetic and pharmacodynamic (PK/PD) studies when using complement inhibitors for the treatment of TA-TMA, owing to the increased catabolic activity post-HSCT. This is of particular importance as it has been demonstrated that suboptimal levels of Eculizumab may lead to treatment failure with a detrimental effect on survival. However, not all centers have access to (PK/PD) studies, which constitutes another obstacle to the successful management of TA-TMA. Finally, the high costs of novel treatments, such as complement inhibitors, cannot be ignored. The exaggerated prices have restricted their use in many countries and, even in those where they are available, they constitute a significant financial burden for healthcare systems. It is then essential to apply reasonable prices for the broad use of effective treatments worldwide.

## Figures and Tables

**Table 1:** Overview of different diagnostic criteria for TA-TMA.

BMT CTN [36]	IWG [37]	Cho et al. ^§^ [17]	Shayani et al. ^±^ [38]	Jodele et al. [14]
≥2 schistocytes per HPF on peripheral smear	≥4% schistocytes in blood	≥2 schistocytes per HPF on peripheral smear	Presence of schistocytes, persistent nucleated RBCs	Presence of schistocytes in peripheral blood or histologic evidence of microangiopathy
Increased LDH ^1^	New increase in LDH ^1^	Increased LDH ^1^	Increased LDH ^5^	Increased LDH ^1^
Renal and/or neurologic dysfunction ^2^	De novo, prolonged or progressive thrombocytopenia ^3^	*De novo*, prolonged or progressive thrombocytopenia ^3^	Prolonged or progressive thrombocytopenia ^3^	*De novo*, prolonged or progressive thrombocytopenia ^3^
Negative Coombs (direct and indirect)	Decreased Hemoglobin or Increased Tx requirements	Negative Coombs (direct and indirect)	Renal impairment ^6^	De novo anemia or anemia requiring Tx support ^7^
	Decreased haptoglobin	Decreased haptoglobin		Absence of coagulopathy
		Normal coagulation assays ^4^		Negative Coombs

^1^ Above upper limit of normal. ^2^ Without other explanations. ^3^ Platelet count < 50 × 10^9^/L or 50% or greater reduction from previous counts. ^4^ Coagulation assays include prothrombin time and activated partial thromboplastin time. ^5^ LDH > ×2 upper limit of normal. ^6^ Renal impairment: serum creatinine clearance > ×1.5 baseline. ^7^ Hemoglobin below the lower limit of normal. ^§^ Used a broad definition of overall-TMA (O-TMA). ^±^ The presence of all 4 criteria listed defines ‘definite’ TMA, whereas the presence of 3/4 criteria defines ‘probable’ TMA. Abbreviations: HPF: high-power field, LDH: lactate dehydrogenase, Tx: transfusion, RBCs: red blood cells.

## Data Availability

Not applicable.
